# Ablative Therapies for Cervical Intraepithelial Neoplasia in Low-Resource Settings: Findings and Key Questions

**DOI:** 10.1200/JGO.18.00093

**Published:** 2018-10-29

**Authors:** Miriam L. Cremer, Gabriel Conzuelo-Rodriguez, William Cherniak, Thomas Randall

**Affiliations:** **Miriam L. Cremer**, Cleveland Clinic, Cleveland, OH; **Gabriel Conzuelo-Rodriguez**, Basic Health International, New York, NY; **William Cherniak**, Bridge to Health Medical and Dental, Toronto, Ontario, Canada; and **Thomas Randall**, Massachusetts General Hospital, Boston, MA.

## Abstract

Barriers to access for cervical precancer care in low-resource settings go beyond cost. Gas-based cryotherapy has emerged as the standard treatment in these areas, but there are barriers to this technology that have necessitated the development and implementation of affordable and portable alternatives. This review identifies knowledge gaps with regard to technologies primarily used in low-resource settings, including standard cryotherapy, nongas-based cryotherapy, and thermoablation. These gaps are addressed using evidence-based guidelines, patient and provider acceptability, long-term obstetric outcomes, and treatment of women with HIV infection. This review highlights the need for prospective studies that compare ablative methods, especially given the increasing use of thermoablation.

## INTRODUCTION

Cervical cancer incidence and mortality have decreased significantly in most developed countries since the 1990s.^1^ However, the burden of disease has remained high in low- and middle-income countries (LMICs), where approximately 80% of the global incidence occurs, with estimated mortality of more than 60%.^2^

Adequate screening followed by treatment of cervical precancer, specifically cervical intraepithelial neoplasia grade 2 and higher (CIN2+), can prevent the development of malignant lesions. Excisional procedures such as loop electrocautery excision procedure (LEEP) and cold knife conization often are unavailable in low-resource settings, and women are treated most often by ablation therapy. Ablation therapy is appropriate for lesions that involve less than 75% of the cervix, do not extend into the endocervical canal or onto the vagina, and are not suggestive of invasive cancer.^3^ This treatment approach often is linked to see-and-treat protocols and can be performed through the following modalities: gas-based (standard) cryotherapy, nongas-based cryotherapy, and thermoablation (sometimes called cold coagulation or thermocoagulation).

Gas-based cryotherapy is the only ablative treatment currently endorsed by the WHO for the treatment of CIN2+ in LMICs.^4^ In many low-resource countries, however, this method has limitations because of the expense of compressed gas and related challenges with procurement and transport.^5,6^ In light of these impediments, a pressing need exists for efficient, affordable, and low-maintenance treatment options for cervical precancer in LMICs.

This review identifies current knowledge gaps with regard to ablation treatments and their use in low-resource settings. Specific areas of focus are the difficulty in standardizing treatment protocols, the acceptability of new technology, long-term obstetric outcomes after treatment, and treatment of women with HIV infection.

## DATA EXTRACTION AND METHODOLOGY

An electronic search was performed in PubMed and Web of Science using three sets of searching algorithms. These consisted of one fixed keyword (cervical intraepithelial neoplasia[MeSH] OR cervical intraepithelial neoplasia[tiab] OR CIN[tiab]) and one variable keyword for each ablative method stated as follows: cryotherapy[MeSH] OR cryotherapy[tiab] for gas-based cryotherapy, nongas cryotherapy[tiab] OR CryoPen[tiab] OR CryoPop[tiab] for nongas-based cryotherapy, and cold coagulation[tiab] OR thermocoagulation[tiab] OR electrocoagulation[tiab] OR thermoablation[tiab] OR thermal ablation[tiab] for thermoablation. Language was set to English and Spanish. The choice of keywords for thermoablation was set to capture the wide variety of terms used in the literature to refer to this procedure. For gas-based cryotherapy, we restricted our search from 2011 to the present. The title and abstract of each publication were reviewed for eligibility, and only original publications that contained quantitative or qualitative data were included. A total of 38 articles for gas-based cryotherapy, 18 for thermoablation, and one for nongas cryotherapy were reviewed for inclusion.

## REVIEW OF CURRENT AND EMERGENT ABLATIVE TECHNOLOGIES

Standard cryotherapy is a gas-based ablation method that has been used to treat CIN2+ since 1964^7^ (Table 1). Many gas-based cryotherapy devices are commercially available that use either compressed carbon dioxide (CO_2_) or nitrous oxide (N_2_O) gas to freeze cervical tissue and cause necrosis. WHO has developed a set of technical specifications for cryosurgical equipment that includes a thorough comparison of devices.^8^ Cure rates range from 77% to 93%, which is similar to cure rates for excisional methods like LEEP.^9^

**Table 1:**
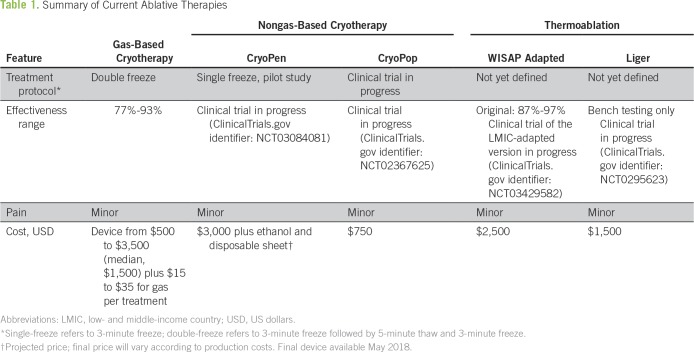
Summary of Current Ablative Therapies

CryoPen (CryoPen, Corpus Christi, TX) is powered by electricity rather than by gas and ablates cervical tissue through the application of a cryoprobe chilled to a freezing temperature of −70°C.^10^ The original CryoPen (developed for use in high-income medical settings) has been adapted for use in LMICs through modifications that improve portability, durability, and affordability.^11^ The LMIC-adapted CryoPen consists of a Stirling cooler built into a toolbox-sized carrying case and an adjoining cryoprobe. The entire device weighs 20 lb, can be carried by hand, and is capable of treating approximately 24 women in an 8-hour day.^12^ CryoPen is currently under study (ClinicalTrials.gov Identifier: NCT03084081).

CryoPop (Jhpiego, Baltimore, MD), an adaptation of standard cryotherapy technology, is designed to convert CO_2_ gas to dry ice. It uses only one-tenth the amount of gas per treatment compared with conventional cryotherapy devices. A current randomized controlled trial (ClinicalTrials.gov Identifier: NCT02367625) in the Philippines currently is comparing CryoPop with standard cryotherapy.

Thermoablation uses heat instead of cold to ablate tissue. It was initially developed to control post-LEEP bleeding but has also been used to treat CIN2+. In a meta-analysis of 13 thermal ablation studies, Dolman et al^13^ estimated a cure rate of 96% (95% CI, 92% to 99%) for CIN1 and 95% (95% CI, 92% to 98%) for CIN2+. Although excisional methods have replaced thermoablation in many high-resource settings, this technique has re-emerged in LMICs as an alternative to cryotherapy. The original WISAP Cold Coagulator device (WISAP Medical Technology, Brunnthal, Germany) consisted of a simple electrical unit with a temperature dial and a probe attached by a cable. The LMIC-adapted device is handheld and operates with electricity or an external battery and currently is being tested in the previously mentioned trial (ClinicalTrials.gov Identifier: NCT03084081). An improved prototype will be tested in an upcoming study (ClinicalTrials.gov Identifier: NCT03429582). Another handheld device, the Liger thermocoagulator (Cure Medical, Lehi, UT) is a portable, battery-powered instrument that is commercially available and also undergoing an NIH-funded trial (ClinicalTrials.gov Identifier: NCT02956239).

Despite the potential benefits of electricity-based technologies, electrical outages are common in rural areas of LMICs, thus hindering their use. However, current LMIC-adapted versions of the CryoPen and thermoablation devices have been developed to run on a car battery or are provided with external battery packs to overcome this problem.

## TREATMENT PROTOCOL AND IMPLEMENTATION

Current WHO guidelines for CIN treatment recommend the following cryotherapy protocol: a double-freeze cycle (3-minute freeze, 5-minute thaw, 3-minute freeze) with CO_2_ or N_2_O gas at or below −20°C. WHO acknowledges that this is a weak recommendation.^3^ The current recommendation for use of the CryoPen is a single 5-minute freeze, and CryoPop uses a double-freeze cycle. Neither technology currently is endorsed by WHO.

Few studies have evaluated alternatives to the double-freeze protocol. Two investigated the use of a single-freeze cycle and the resulting depth of cervical tissue necrosis. Adepiti et al^14^ compared the effectiveness of double-freeze versus single-freeze N_2_O cryotherapy and used a depth of necrosis benchmark of ≥ 4.8 mm, which was established from prior preclinical studies. With a double-freeze protocol, the benchmark was met in 87% and 73% of patients (anterior and posterior lip, respectively); conversely, among patients who underwent a single-freeze cycle, the benchmark was met in only 37% and 20%, respectively. Cremer et al,^15^ however, found that a 5-minute single freeze with N_2_O achieves a mean depth of necrosis that is noninferior to that achieved with a double freeze. Mariategui et al^16^ compared the effectiveness of both gases (CO_2_ and N_2_O) and found that maximum depth of necrosis is greater with N_2_O (5.3 mm) than with CO_2_ (3.4 mm). WHO guidelines state that either gas is acceptable.^3^

No WHO guidelines exist with regard to thermoablation. Furthermore, there is no consensus in the literature about the optimal thermoablation temperature, application time, or number of applications (Table 2). A limitation of all of the thermoablation studies is that cure rates are based on cytology with or without colposcopy findings rather than on biopsy results. The sensitivity of cytology is estimated to be approximately 50%, thus making this a major limitation in estimating cure rates.^17^

**Table 2:**
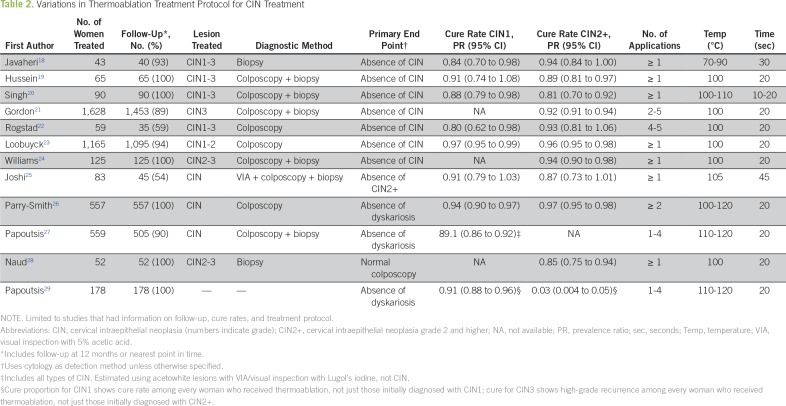
Variations in Thermoablation Treatment Protocol for CIN Treatment

Available research suggests that a 20-second thermoablation regimen may be inadequate on the basis of depth of necrosis. Although the clinical significance of depth of necrosis is uncertain, it is a useful parameter for protocol comparisons. For example, Haddad et al^30^ reported a mean depth of tissue necrosis of 2.6 mm after treatment with a single application of thermoablation at 100°C for 20 seconds, whereas the depth of necrosis reached 3.5 mm among women who received treatment at 120°C for 30 seconds. Chen et al^31^ used chicken breasts as a proxy for human cervical tissue and found a mean depth of necrosis of 3.75 mm after thermoablation with the Liger device at 100°C for 20 seconds, yet the mean depth of necrosis increased to 3.9 mm and 4.7 mm after using 30- and 40-second applications, respectively. The standardization of the optimal thermoablation protocol is essential to yield reproducible results and successful interventions. Upcoming research should address the best protocol to follow, particularly for new technologies and thermoablation.

## ACCEPTABILITY BY PROVIDERS AND PATIENTS

WHO’s strongest recommendation about cryotherapy (ie, the one with the most evidence) is that midlevel providers can perform the procedure.^3^ Ablative therapies can be administered by many different levels of trained providers, which include but are not limited to physicians. In many implementation studies on cryotherapy^5,6,32-34^ and thermoablation,^35,36^ nonphysician health professionals provided treatment. To our knowledge, however, no studies have specifically assessed provider acceptability.

Some studies have explored patient acceptability and morbidity associated with cryotherapy. Watery discharge is the most common adverse effect followed by mild abdominal pain.^32-34,37^ Three studies assessed patient satisfaction after treatment. Adefuye et al^37^ reported that 98.2% of 220 women believed that the procedure had been as they expected, and 95% said that they would recommend cryotherapy to other women. Lewis et al^33^ found 100% satisfaction with cryotherapy among 22 women surveyed at their 3-month follow-up visit. This result reflects the responses of only those who returned for follow-up, however, which suggests possible attrition bias. Phongsavan et al^34^ found that all 113 women treated with cryotherapy reported being satisfied or very satisfied with the treatment.

A few studies evaluated thermoablation acceptability in terms of adverse events.^28,38^ In a sample of 52 women, Naud et al^28^ reported pain/cramps (79%) as the most common adverse event followed by heat sensation in the vagina (25%). Viviano et al^38^ found that although 95.5% of the 110 women they studied experienced some degree of pain during therapy when no analgesia was used, the mean pain rating was 3 of 10 (standard deviation, 1.6) on the visual analog scale, which indicates mild or moderate pain. A study by Duncan et al^39^ that compared two analgesics (prilocaine and felypressin) for pain relief during the procedure concluded that both drugs are efficient at reducing pain. Nevertheless, the authors concluded that pain is well tolerated and that most patients do not require analgesia.

Preliminary analyses have shown that both CryoPen and standard cryotherapy are well tolerated by women. Alfaro et al^40^ reported no difference in pain experienced during standard cryotherapy versus CryoPen treatment. Additional research should investigate provider attitudes toward treatment using both qualitative and quantitative approaches. Similarly, patients’ acceptability must be quantified using methods such as satisfaction surveys that include pain experienced during the procedure and other adverse effects.

## LONG-TERM OBSTETRIC OUTCOMES

Given that many women treated for CIN are of reproductive age, effects on obstetric outcomes are a significant concern. Overall, women who undergo any CIN treatment are at greater risk for preterm birth and other perinatal morbidity compared with the general population with no treatment.^41^ Very little research has been done on obstetric outcomes after ablative treatment. Two studies showed an association between excisional treatment and preterm birth.^42,43^ Although some evidence suggests that there are fewer risks after ablative therapies compared with excisional therapy, data on individual methods are lacking.

Additional research is essential to expand our knowledge about obstetric outcomes after ablative therapies, especially in LMICs. Prospective trials would be ideal, but they are difficult to perform because of the recruitment and follow-up that would be needed to detect small differences of relatively rare outcomes. Additional methodological problems arise from underpowered studies, including selection bias and inadequate confounding measurement and control. Retrospective data, despite their limitations, may provide insight into population-based data.

Future studies also should consider the underlying pathophysiology and conditions that may contribute to obstetric outcomes among women treated for CIN. These influences may be related to human papillomavirus (HPV) infection, the host immune response, the distortion of cervical physiology by the treatment, or a combination of factors. Accurate histologic confirmation of treated lesions and appropriate follow-up data can help to resolve these gaps in knowledge. In this sense, postmarket surveillance and registries may yield valuable information to answer the question about the best method to treat women of reproductive age.

## WOMEN WITH HIV INFECTION

Several studies have reported that women with HIV infection are at greater risk of having multiple high-risk HPV types associated with the development of CIN2+ compared with women without HIV.^44,45^ No standard guidelines exist for cervical precancer treatment of women with HIV infection in LMICs, but some studies have examined the efficacy of ablative therapies in this population (Table 3).

**Table 3:**
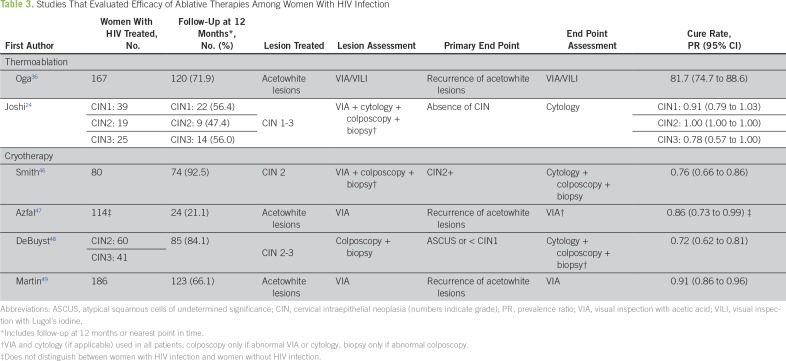
Studies That Evaluated Efficacy of Ablative Therapies Among Women With HIV Infection

The major methodological limitations of these studies concern patient and outcome definition and assessment, which complicate the interpretation of cure rates. Although estimated cure rates of ablative therapies are above 75%, this is lower than that estimated among women without HIV infection.^9^ Follow-up is inconsistent across studies, and attrition rates are high in many. Only one published study compared LEEP versus cryotherapy and found no difference in the 12-month cumulative incidence of CIN2+.^46^

Additional research into ablative therapy is needed for women with HIV infection because many questions remain to be answered. Do existing screening protocols address the needs of an HIV population? How do the various ablative therapies compare with one another in this population? When should patients come for their follow-up visit, and what should be done at this visit? It is known that the highest quality data for cure rates come from biopsy-proven clearance of disease, but currently, no standard method to assess cure exists, and a high-risk HPV DNA test is a promising alternative given its high sensitivity (90%), negative predictive value (88%), and optimal reproducibility. To ensure robust data, studies should aim for at least an 80% compliance rate with the follow-up visit.

## DISCUSSION

Current evidence suggests that cryotherapy and thermoablation are effective treatment methods for CIN and that nongas-based cryotherapy technologies are promising alternatives under development. However, no clear guidelines currently exist for treatment protocols that use thermoablation methods. To our knowledge, no randomized clinical trials have compared these ablative therapies directly with one another. Such studies will be a crucial step to validate the usefulness of any method whether it is widely used but untested or a recent innovation.

Few studies have addressed acceptability by both providers and patients of the various ablative therapies. In low-resource settings, it is especially important that treatment be provided by medical personnel who are not physicians. Medical staff in these settings are consistently overwhelmed by treatment demand; therefore, nonphysician providers must be comfortable with applying treatment. Knowledge gained from future studies that evaluate both patient and provider perspectives in terms of treatment modality and overall care would be useful.

A systematic review by Sauvaget et al^50^ showed that many places have implemented successful cryotherapy programs by using midlevel providers. Although this idea may be generalized to other ablative therapies, the identification of best practices and treatment protocols is essential to reduce subjective assessment and potential errors.

Prospective studies that evaluate long-term obstetric and reproductive outcomes are needed as ablative methods become more widely available in LMICs and may be preferable to excisional therapy for women who plan to become pregnant after treatment. Women with HIV infection are also an important subgroup to consider given the higher prevalence of HIV in LMICs and an increased risk for high-grade CIN. An investigation in these cohorts will help to evaluate residual disease as well as recurrence, reinfection, and progression rates. In addition, they will help to identify the optimal follow-up strategy after treatment.

That the successes of early detection and treatment of cervical precancer experienced in developed countries are not enjoyed by the countries where these interventions are now most needed is unacceptable. Evidence suggests that treatment is most successful when coupled with screening in a single visit. However, faulty equipment, supply shortages, and lack of trained personnel are still significant limitations to this screen-and-treat approach in LMICs. Research and development of new technologies amenable to LMICs must continue to increase access to effective treatment of cervical precancer in these settings.
